# Immunomodulatory Efficacy of Standardized* Annona muricata* (Graviola) Leaf Extract via Activation of Mitogen-Activated Protein Kinase Pathways in RAW 264.7 Macrophages

**DOI:** 10.1155/2016/2905127

**Published:** 2016-12-21

**Authors:** Goon-Tae Kim, Nguyen Khoi Song Tran, Eun-Hye Choi, Yoo-Jeong Song, Jae-Hwi Song, Soon-Mi Shim, Tae-Sik Park

**Affiliations:** ^1^Department of Life Science, Bokjung-dong, Sujung-gu, Sungnam-si, Gyeongg-do 461-701, Republic of Korea; ^2^Department of Food Science and Technology, Sejong University, 98 Gunja-dong, Gwangjin-gu, Seoul 143-747, Republic of Korea

## Abstract

*Annona muricata*, commonly known as Graviola, has been utilized as a traditional medicine to treat various human diseases. The aim of this study was to examine the immune-enhancing activity of Graviola leaf extracts in RAW 264.7 macrophage cells. Active ingredients in Graviola leaf extracts (GE) were identified as kaempferol-3-O-rutinoside and quercetin-3-O-rutinoside by LC-MS/MS. When treated with steam or 50% ethanol GE, cell morphology was altered due to initiation of cell differentiation. While the cell viability was not altered by the steam GE, it was reduced by the ethanol GE. Both steam and ethanol GE induced the transcriptional expression of cytokines, including tumor necrosis factor-*α* (TNF-*α*) and interleukin-1*β*, but only the steam extract upregulated inducible nitric oxide synthase (iNOS). In consistence with mRNA expression, the production of TNF-*α* and nitrite was elevated by both steam and ethanol extracts of Graviola leaves. This is mainly due to activation of mitogen-activated protein (MAP) kinase signaling pathways. These results suggest that Graviola leaves enhance immunity by activation of the MAP kinase pathways. These bioactive properties of Graviola indicate its potential as a health-promoting ingredient to boost the immune system.

## 1. Introduction

Medicinal natural products have been traditionally applied for treatment of diseases and their active constituents have been studied for elucidation of their mechanisms of action [1, 2].* Annona muricata* L. is a medicinal plant, commonly known as Graviola, Soursop, or Guabana. Graviola is a tropical evergreen fruiting tree, which belongs to the Annonaceae family [3, 4]. All parts of the Graviola tree are known to be effective against various human diseases such as cancer and parasitic infections. In particular, Graviola leaves were found to be effective against cystitis, diabetes, headache, insomnia, and inflammation [5, 6]. In South America and Africa, Graviola leaves have been applied as an ethnomedicine to treat cancer. Besides its medicinal uses, Graviola fruits are used for development of various food products including beverages, candy, and syrups [7].

These traditional pharmacological uses of Graviola have evoked an interest for further elucidation of its effective components. Among various constituents in Graviola, acetogenins have been applied for cancer therapy. Annonaceous acetogenins are composed of 35- or 37-carbon long-chain fatty acids that form the polyketide skeleton. These acetogenins, derived from seeds, leaves, and pericarp have cytotoxicity against carcinoma cells of prostate, pancreas, and breast [6, 8, 9]. Despite their anticancer efficacy, some acetogenins in Graviola leaves are known to be highly cytotoxic [10]. In addition, chronic consumption of Graviola juice containing annonaceous acetogenins aggravates cerebral tau phosphorylation in mice and contributes to development of neuropathological changes observed in Parkinson's disease [11, 12]. Another important attribute about Graviola is its high content of antioxidant compounds. Phenolic acids and flavonoid glycosides in Graviola leaves are known to have potent antioxidant activities [13]. Recently, we have reported that the total phenolic acid and total flavonoid contents were present in the steam and ethanol extracts of Graviola leaves in high concentrations, which is associated with their free radical-scavenging effects [14]. Additionally, the ethanol extract of Graviola leaves was found to upregulate the expression of superoxide dismutase-1 (SOD-1) and nuclear factor erythroid-2-related factor-2 (Nrf-2), which contributed to its reactive oxygen species-reducing activity.

The adaptive immune system is a defense mechanism to prevent the invasion by microorganisms or exogenous materials. Natural products, derived mainly from plants have been widely studied in immune-related diseases [15]. Stimulation of the immune system is particularly important in immunocompromised patients, such as those after surgery and HIV-infected and senile people, to prevent the occurrence of secondary infections. Besides its use as supplemental food, plant-derived products are being consumed as herbal medicines to prevent progression of chronic diseases including cancer and infections.

These previous reports prompted us to question whether the Graviola leaf extracts (GE) modulate the immune response aroused by inflammatory mediators. The findings that oral intake of ethanol GE significantly reduced carrageenan-induced edema in rat paws support our hypothesis [16]. However, the effects of GE on modulation of immune responses induced by inflammatory mediators have not been elucidated. In this study, we examined the role of GE in macrophage activation and regulation of cytokines production.

## 2. Materials and Methods

### 2.1. Preparation of Standardized Graviola Leaf Extract

Graviola leaves, grown in the Philippines, were obtained from Graviola and Nature Company (Korea). For the steam extract, air-dried Graviola leaves (92.5 g) were heated at 141°C for 6 h and then indirectly heated using boiled water (100°C) for 1 h. They were collected and condensed to powder by a rotary vacuum evaporator at 55°C at a speed of 70 rpm. The final extract powder was kept at −20°C until use for analysis. For the 50% ethanol extract, air-dried Graviola leaves (63.7 g) were blended with ethanol/distilled water (1 : 1, v/v) and then kept in a shaking water bath (BF-30SB; Biofree Co. Ltd., Seoul, Korea) at 55°C at a speed of 70 rpm for 24 h. It was filtered through an 8.0 *μ*m filter paper (Whatman Inc., Piscataway, NJ, USA) and concentrated using the same method used for the steam extract. It was kept at −80°C for 8 h and then dried in a freeze-drier for 5 days. It was then stored at −20°C until use for analysis. Bioactive components in standardized GE were analyzed by LC-MS/MS as reported previously [14].

### 2.2. Chemical Fingerprint Analysis of Graviola Leaf Extract by Using UPLC/ESI-MS Methods

Bioactive components in the extracts were analyzed by using an ultra-performance liquid chromatography/electrospray ionization-mass spectrometry (UPLC/ESI-MS) coupled with Accela photo diode array (PDA) detector, Accela auto sampler, and Accela pump (Thermo Fisher Scientific, Waltham, MA, USA). The analytes were separated on a Zorbax Eclipse XDB-C18 (250 mm × 4.6 mm, 5 *μ*m, Agilent technologies) column. Detailed analytical conditions were used as described in a previous study [14]. In detail, the mobile phases used were solvent A (1% formic acid in water, HPLC grade, Sigma-Aldrich) and solvent B (acetonitrile, HPLC grade, Sigma-Aldrich) with gradient elution as follows: 95–5% A at 0–60 min. The analysis was carried out at a flow rate of 1.0 mL/min. UV wavelength was measured at 340 nm. ESI-MS spectra were acquired in positive ion mode. For the full-scan MS analysis, the spectra were recorded in the range of mass to charge ration (*m*/*z*) 100–800. The source parameters were as follows: electrospray capillary voltage = 30 V, capillary temperature = 200°C, sheath gas flow = 30 arb, and auxiliary gas flow = 2.0 arb. The stability of extracts was evaluated using other three samples.

### 2.3. Cell Culture and Cell Viability Assay

RAW 264.7 cells were obtained from the American Type Culture Collection (ATCC; Manassas, VA, USA). Cells were cultured in high glucose Dulbecco's Modified Eagle's Medium (DMEM) supplemented with 10% fetal bovine serum (FBS), 100 U/mL penicillin, and 100 *μ*g/mL streptomycin under 5% CO_2_-humidified atmosphere at 37°C. Cell viability was measured with the help of a cell proliferation assay kit (Welgene Inc., Korea), using 2,3-bis-(2-methoxy-4-nitro-5-sulfophenyl)-2H-tetrazolium-5-carboxanilide (XTT).

### 2.4. RNA Isolation and Real-Time RT-PCR

RAW 264.7 cells were treated with 0, 50, 100, and 200 *μ*g/mL GE for 8 h after 8 × 10^5^ cells/well were seeded into a 6-well plate and incubated for 24 h. Total RNA was isolated from RAW 264.7 cells using RNA Extraction Kit (Intron Biotechnology Inc., Korea) according to the manufacturer's instructions. cDNA was synthesized in a PCR Thermal Cycler (TaKaRa, Japan), using the iScript cDNA synthesis Kit (Bio-Rad Laboratories, Hercules, CA, USA). PCR conditions were 25°C for 5 min, 42°C for 30 min, and 8°C for 5 min and then kept at 4°C for maintenance. Real-time PCR was performed in Step-One Plus real-time PCR system (Applied Biosystems, Carlsbad, CA, USA), using primers and SYBR Green Master Mix (TaKaRa). Real-time PCR conditions were initially maintained at 95°C for 5 min, followed by 40 cycles of 95°C for 15 sec, 60°C for 15 sec, 72°C for 45 sec, 95°C for 15 sec, and 60°C for 1 min. The sequences of the primers used in this study were as follows: IL-1*β*, forward-ATG GCA ACT GTT CCT GAA CTC AAC T, reverse-GTG CTG CCT AAT GTC CCC TTG AAT C; IL-6, forward-AGT TGC CTT CTT GGG ACT GA, reverse-CAG AAT TGC CAT TGC ACA AC; COX-2, forward-CCC CCA CAG TCA AAG ACA CT, reverse-CTC ATC ACC CCA CTC AGG AT; iNOS, forward-AAT GGC AAC ATC AGG TCG GCC ATC ACT, reverse-GCT GTG TGT CAC AGA AGT CTC GAA CTC; TNF*α*, forward-ACA AGC CTG TAG CCC ACG, reverse-TCC AAA GTA GAC CTG CCC; *β*-actin, forward-GAC GTT GAC ATC CGT AAA G, reverse-CAG TAA CAG TCC GCC T. Expression of the genes was normalized to that of *β*-actin.

### 2.5. Western Blot Analysis

RAW 264.7 cells were treated with various concentrations of GE for 30 min after 8 × 10^5^ cells/well were seeded into a 6-well plate and incubated for 24 h. Cells were homogenized in a cell lysis buffer containing 10 mM Tris (pH 7.8), 1 mM EDTA, 150 mM NaCl, phosphatase inhibitor, and protease inhibitor. The lysates were then centrifuged to remove cell debris and unbroken cells. Proteins were quantified using Protein Assay kit (Bio-Rad Laboratories) and the absorbance was measured at 750 nm. Thirty micrograms of the assayed protein was separated by using 10% acrylamide gel and then the proteins in the gel were transported to a PDVF membrane. The protein-specific primary antibody was attached and the mouse-specific or rabbit-specific secondary antibodies were then added. Proteins of interest were visualized by Enhanced Chemiluminescence kit (Bio-Rad Laboratories) using a Chemiluminescence imaging equipment (Vilber Lourmat, France). Primary antibodies against pJNK, JNK, pERK1/2, ERK1/2, pp38, and p38 (Cell Signaling Technology, Danvers, MA, USA) and *α*-tubulin (Sigma-Aldrich, St. Louis, MO, USA) were used.

### 2.6. Nitrite Quantification Assay

RAW 264.7 cells were treated with various concentrations of GE for 24 h after 8 × 10^5^ cells/well were seeded into a 6-well plate and incubated for 24 h. Cells were also treated with 1 *μ*g/mL lipopolysaccaride (LPS). Culture media, isolated, and the concentration of nitrite, an indicator of nitric oxide (NO) synthesis, were measured by using Griess reagent (1% sulfanilic acid in 5% phosphoric acid and 0.1%* N*-ethylenediaminedihydrochloride; Molecular Probes, Waltham, MA, USA). The supernatant of the treated cell culture media (150 *μ*L) was mixed with Griess reagent (20 *μ*L) and deionized water (130 *μ*L) in a 96-well plate and incubated for 30 min. The absorbance was then measured at 548 nm in a microplate reader. NO concentration was determined using a dilution of sodium nitrite as a standard.

### 2.7. Measurement of Cytokine (IL-1*β* and TNF-*α*) Levels

RAW 264.7 cells were treated with various concentration of GE for 24 h after 8 × 10^5^ cells/well were seeded into a 6-well plate and incubated for 24 h. LPS (1 *μ*g/mL) served as a positive control. The concentration of TNF-*α* and IL-1*β* was measured using ELISA kit (Enzo Life Sciences, Ann Arbor, MI, USA). Samples were put in a microtiter plate to which the monoclonal antibody was attached and then shaken at a speed of 500 rpm for 2 h. Polyclonal antibody was added to the wells after four washing cycles with the washing buffer. The conjugate solution was added to each well followed by a washing cycle. After 30 min, the stop solution was added and the absorbance was measured at 450 nm using a microplate reader (Infinite; Tecan, Switzerland). Cytokines concentration was determined using a dilution of kit's standard solution.

### 2.8. Statistical Analysis

All results are expressed as mean ± the standard error of mean (SEM). At least three independent experiments were performed. Results were analyzed by using Student's* t*-test and* p* value less than 0.05 was considered statistically significant.

## 3. Results

### 3.1. Standardized Graviola Leaf Extract (GE) Containing Flavonols

**Figure 1:**
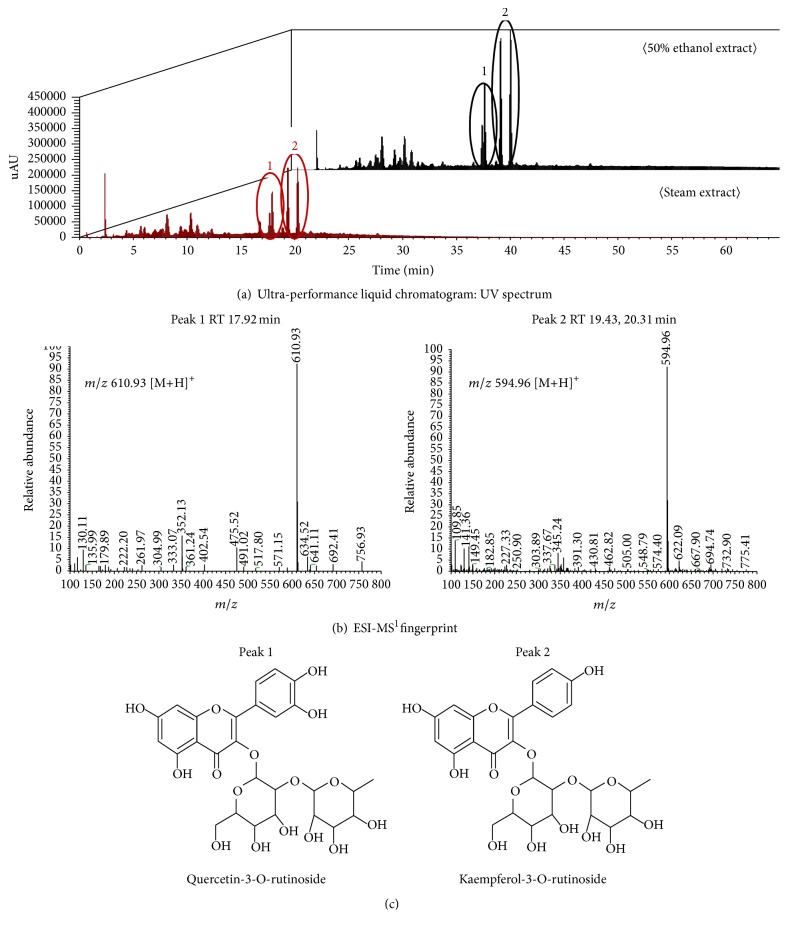
Identification of major components in the steam and 50% ethanol GE. GE were analyzed by UPLC/ESI-MS and the absorbance was measured at 340 nm (a). Major peaks were fingerprinted by identifying major *m*/*z* (b) and found to be quercetin-3-O-rutinoside and kaempferol-3-O-rutinoside (c).

To find out the major bioactive compounds in GE, we analyzed the components of the steam and 50% ethanol GE by UPLC/ESI-MS. As shown in the chromatograms (Figure 1(a)), two major peaks in standardized GE were eluted at retention time of 17.92 (peak 1) and 19.43 min (peak 2). Chemical fingerprints analyzed by ESI-MS with positive ion mode identified 610.93 and 594.96 *m*/*z* for peak 1 and peak 2, respectively (Figure 1(b)). Based on the structural identity, the standardized GE contains two components of flavonols such as quercetin-3-O-rutinoside and kaempferol-3-O-rutinoside (Figure 1(c)). Quercetin-3-O-rutinoside has disaccharide binding with hydroxyl group of quercetin and its molecular weight is 610.52 g/mole. Another major flavonol identified in GE was kaempferol-3-O-rutinoside which forms a glycosidic bond with quercetin-3-O-rutinoside and its molecular weight is 594.52 g/mole.

The two bioactive components in GE were evaluated with three duplicates extract batch during intraday in order to confirm the accuracy of amount of bioactive components in the extract by LC-MS/SM. The value of quercetin-3-O-rutinoside was 614.42, 608.38, and 623.61 *μ*g per g dry weight in the steaming GE. Their Relative Standard Deviation (RSD) of accuracy was 1.02, 1.03, and 1.00%, indicating that they are within 5% of RSD range. The amount of kaempferol-3-O-rutinoside was 690.57, 706.97, and 703.83 *μ*g per g dry weight that were calculated into 1.83, 1.78, and 1.79% of RSD, respectively. In contrast, the value of quercetin-3-O-rutinoside was 1428.82, 1431.5, and 1475.06 *μ*g per g dry weight in the 50% ethanol GE. Their Relative Standard Deviation (RSD) of accuracy was 1.48, 1.48, and 1.44%. The amount of kaempferol-3-O-rutinoside was 1658.27, 1720.96, and 1686.32 *μ*g per g dry weight, which were calculated into 1.55, 1.49, and 1.52% of RSD, respectively. The results from the current study confirmed that the steaming and 50% ethanol GE containing two flavonols were standardized. They were used for further study on evaluating immunomodulatory efficacy.

### 3.2. GE Alters Macrophage Morphology and Induces Differentiation

**Figure 2:**
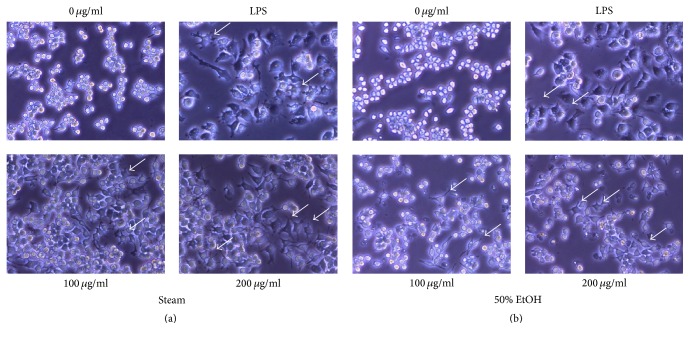
Altered morphology of RAW 264.7 cells after GE treatment. Cells were treated with the steam (a) or 50% ethanol (b) GE for 24 h at various concentrations.

We questioned whether GE activates macrophage differentiation. When macrophages are exposed to endotoxins, such as LPS, a morphological change occurs, which is the first step to activate an inflammatory response [17]. RAW 264.7 macrophage cells were incubated with various concentrations of the steam or ethanol GE, respectively, and cell morphology was observed (Figure 2). When cells were cultured in an empty medium, most of the cells displayed a circular morphology and normal proliferation. However, when cells were treated with LPS, the cell morphology was changed to an extended form and proliferation was suppressed. In the presence of the steam or ethanol GE, the cell morphology was significantly altered in a similar manner as LPS-treated cells (arrows in Figures 2(a) and 2(b)).

**Figure 3:**
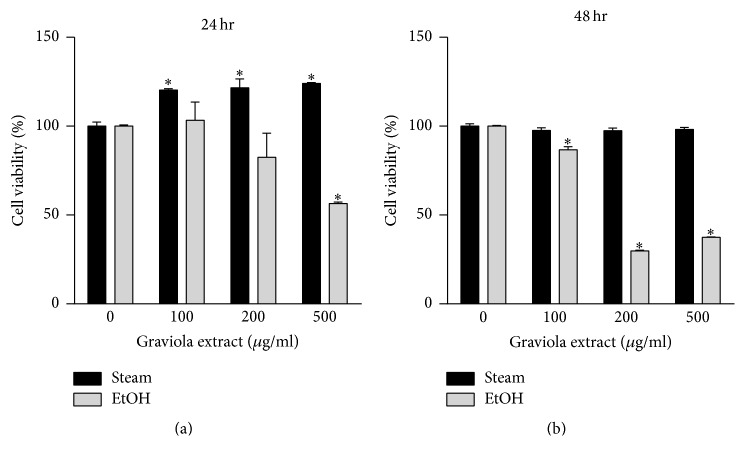
Cytotoxicity of GE in RAW 264.7 cells. Cells were treated with steam or 50% ethanol GE at various concentrations for 24 h (a) or 48 h (b). Cell viability was measured by XTT assay after incubation. The results were expressed as a percentage relative to the vehicle-treated control. The data were expressed as mean ± SEM. ^*∗*^ *p* < 0.05 versus vehicle-treated control.

Moreover, we investigated whether GE affects the cell viability to determine its cytotoxicity. RAW 264.7 cells were incubated with the steam or 50% ethanol GE for 24 and 48 h, respectively. The steam GE did not affect the cell viability but rather increased cell proliferation after 24 h of incubation (Figure 3(a)) and the cell viability remained the same after 48 h incubation (Figure 3(b)). In contrast, the 50% ethanol GE decreased the cell viability after both 24 and 48 h incubation in a dose- and time-dependent manner (Figures 3(a) and 3(b)). These results suggest that both the steam and 50% ethanol GE induce macrophage activation but only the steaming GE does not have any cytotoxic effect on RAW 264.7 cells.

### 3.3. GE Activates Production of Cytokines and NO

**Figure 4:**
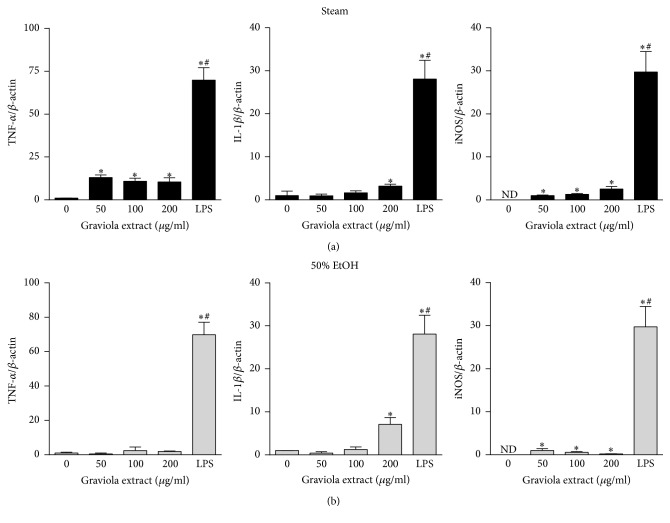
Regulation of expression of cytokines and iNOS by GE. RAW 264.7 cells were treated with the steam (a) or 50% ethanol (b) GE at various concentrations for 24 h. Total RNA was extracted and the expression was measured by real-time PCR. The data were expressed as mean ± SEM. ^*∗*^ *p *< 0.05 versus vehicle-treated control. ^#^ *p* < 0.05 versus GE-treated group. ND, not detected.

**Figure 5:**
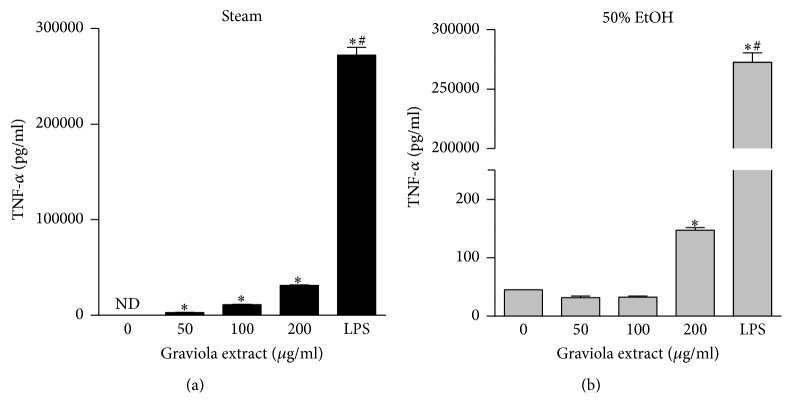
Regulation of TNF-*α* production in RAW 264.7 cells. Cells were treated with the steam (a) or 50% ethanol (b) GE at various concentrations for 24 h. The culture media were collected and cytokine concentrations were measured by ELISA. The data were expressed as mean ± SEM. ^*∗*^ *p* < 0.05 versus vehicle-treated control. ^#^ *p *< 0.05 versus GE-treated group.

**Figure 6:**
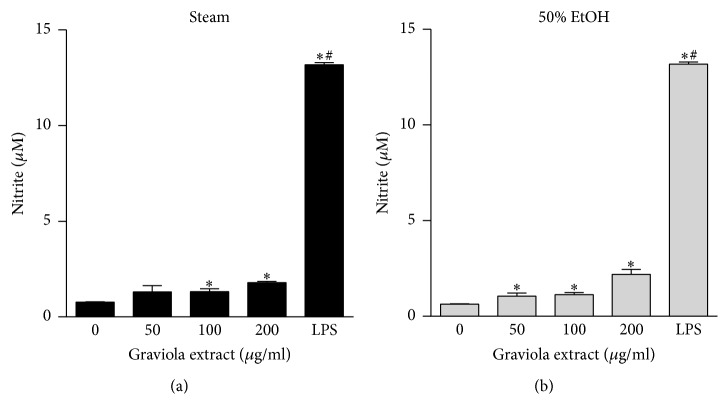
Effect of GE on nitrite production in RAW 264.7 cells. Cells were treated with the steam (a) or 50% ethanol (b) GE at various concentrations for 24 h. The culture media were collected to measure nitrite level using Griess reagent. The data were expressed as mean ± SEM. ^*∗*^ *p* < 0.05 versus vehicle-treated control. ^#^ *p* < 0.05 versus GE-treated group.

Our findings that GE induced macrophage activation led us to investigate its effect on the expression of cytokines and inducible nitric oxide synthase (iNOS). When the macrophages encounter infectious bacteria or endotoxins, their morphology is altered (Figure 2) and production of cytokines is activated due to activation of inflammatory signaling pathways. NO is synthesized in macrophages via the reaction converting l-arginine to l-citruline by iNOS [18]. NO is involved in a defense mechanism against cytosolic microorganisms and acts as a signaling molecule to mediate vasoconstriction [19]. We treated RAW 264.7 cells with various concentrations of GE and measured the mRNA expression of TNF-*α*, IL-1*β*, and iNOS by real-time PCR. The steam GE upregulated the transcriptional expression of TNF-*α*, IL-1*β*, and iNOS (Figure 4(a)). In addition, the 50% ethanol GE upregulated IL-1*β* and iNOS but only a slight increase occurred in TNF-*α* which was statistically not significant (Figure 4(b)). However, the steam GE upregulated cytokines and iNOS expression to a lesser degree compared to LPS. To verify this, RAW 264.7 cells were treated with GE and the culture media were collected to measure cytokine concentrations. In consistence with the transcriptional upregulation, the levels of TNF-*α* (Figure 5) and nitrite (Figure 6) were elevated by the steam and 50% ethanol GE. IL-1*β* was not detected in our current experimental setting. These results indicate that the steam GE activates the transcriptional expression of cytokines and iNOS; however, the 50% ethanol GE has less effect on the expression of those genes.

### 3.4. GE Activates Mitogen-Activated Protein Kinase (MAPK) Pathway

**Figure 7:**
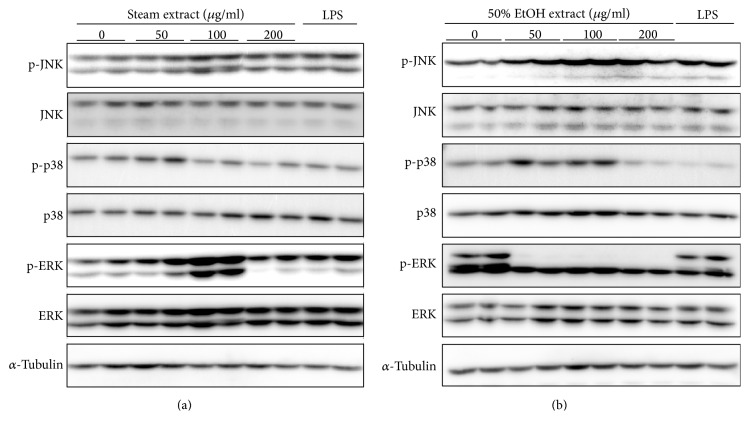
RAW 264.7 cells were treated with either steam (a) or 50% ethanol (b) extract at various concentrations for 30 min. Cells were harvested and protein extracts were analyzed by immunoblotting analyses.

Bacterial and viral infections are recognized by toll-like receptor- (TLR-) 4 on the cell surface which activates inflammatory response as a defense mechanism [20]. The mitogen-activated protein (MAP) kinases are intermediate proteins that mediate inflammatory signals by phosphorylation and serve to modulate macrophage activation and differentiation. Activation of MAPK including ERK, JNK, and p38 leads to upregulation of proinflammatory mediators and activation of cytokines production. We investigated whether GE-induced macrophage activation, represented by morphology alteration and increased production of cytokines and nitrites, is mediated via activation of MAPK signaling pathways. RAW 264.7 cells were incubated with various concentrations of the steam and 50% ethanol GE for 30 min and the degree of phosphorylation of MAPK was examined by western blotting. The steam GE increased the levels of pJNK and pERK at a concentration of 100 *μ*g/mL and pp38 at a concentration of 50 *μ*g/mL (Figure 7(a)). When the cells were treated with the ethanol GE, the levels of pJNK and p38 were elevated (Figure 7(b)). However, p44 (upper band of p-ERK1) phosphorylation was completely blocked. Notably, the levels of phosphorylated MAPK were even higher than those in LPS-treated cells. These results suggest that GE activates MAPK signaling, which is associated with macrophage activation in part.

## 4. Discussion

Health-promoting effects of herbal products have drawn attention to their therapeutic efficacy. Herbal medicines have been traditionally used to alleviate the symptoms of various chronic diseases. However, their mechanisms of action have not been elucidated in most cases. Immunostimulatory efficacy has been reported for products derived from ginseng, echinacea, and astragalus [21, 22]. These medicinal plants can be applied to recover the host defenses in immunocompromised patients. Among these medicinal plants, Graviola leaf has been utilized as a traditional medicine for various diseases including infections, headache, insomnia, and diabetes [5, 6]. In this study, we demonstrated that (1) GE altered cellular morphology and induced macrophage activation; (2) GE activated the gene expression of cytokines and iNOS thus increased their cellular levels; (3) GE increased MAPK phosphorylation.

Graviola is an evergreen fruiting tree. While the fruit is traditionally used to alleviate the symptoms associated with neuralgia, arthritis, diarrhea, dysentery, fever, malaria, parasites, rheumatism, skin rashes, and worms, the leaves are believed to have antirheumatic and neuralgic effects [4]. Among various bioactive components in Graviola, annonaceous acetogenins have antiproliferative effects and thus possess potential anticancer properties [23, 24]. The ethyl acetate extract of Graviola leaves inhibited the proliferation of A549 cells and human breast cancer cells by arresting the cell cycle and inducing apoptosis [5, 25]. Additionally, acetogenins displayed antioxidant activity and are considered as a therapeutic candidate for treatment of chronic diseases initiated by oxidative stress [6, 26, 27]. Besides, Graviola leaf contains various phytoconstituents such as alkaloids, flavonol triglycosides, phenolics, cyclopeptides, and essential oils [6, 13, 24]. Recently, we found that Graviola contains high concentrations of rutin, kaempferol-rutinoside, and vitamin U and effectively scavenged peroxyl and nitrogen radicals [14]. In addition, Graviola reduced the production of reactive oxygen species (ROS).

The findings that GE has high contents of phenolic acids and flavonoids prompted us to investigate its probable proinflammatory effects on macrophage cells. Ishola et al. reported that Graviola fruit reduced carrageenan-induced rat paw edema and xylene-induced inflammatory edema [28]. Surprisingly, GE treatment of RAW 264.7 cells led to macrophage activation and altered morphology. As a result, transcriptional upregulation of inflammatory genes and increased production of cytokines and NO occurred. These conflicting results were probably due to endotoxin-like components in GE and TLR4-mediated activation of innate immune signaling pathways. It is worth noticing that the degree of upregulation and increased cytokine production was less than those induced by LPS. The reason for this is not clear but probably other components in GE may have an antagonizing anti-inflammatory effect against TLR-4-activating components, which can explain the lower efficacy of GE when compared to that of LPS. The major component in GE that induces macrophage activation and activates innate immune responses requires further study and the underlying mechanism should be elucidated.

When an exogenous microorganism invades a vertebrate host, the innate immune system is the first defense line against pathogens. This defense mechanism is mainly mediated by macrophages and dendritic cells. TLR4 on the surface of mammalian cells binds to the antigen on the invading microorganism, which initiates a signaling cascade to activate the immune system. In addition, phosphorylation of MAP kinases including JNK, ERK, and p38 induces innate immune response. We found that both the steam and 50% ethanol GE increased phosphorylation of JNK and p38. While the steam GE increased phosphorylation of p44 and p42 (ERK1/2), the 50% ethanol GE completely blocked the phosphorylation of p44 (ERK1). This finding indicates that regulation of ERK is differently modulated by the steam and 50% ethanol GE. In particular, the cytotoxic effect of the ethanol GE on RAW 264.7 cells (Figure 2) is indicative of this different mode of action. Interestingly, the degree of MAP kinase phosphorylation by GE is higher than those of LPS. This implies that GE is more effective in activating the innate immune signaling pathways than LPS but the interacting components may reduce this effect.

## 5. Conclusion

In conclusion, our findings suggest that GE can activate the innate immune system by inducing macrophage activation and production of cytokines and NO in mouse macrophage RAW 264.7 cells via activation of MAP kinases. These results indicate that GE has immunostimulatory potential and can be applied to boost the innate immune system in immunocompromised patients. In addition to its high contents of bioactive components and ROS-scavenging capacity, the immune-boosting ability of Graviola can be applied for the development of health-promoting functional foods.

## Abbreviations

SOD1: Superoxide dismutase-1
Nrf-2: Nuclear factor erythroid-2-related factor-2
GE: Graviola leaf extracts
XTT: 2,3-Bis-(2-methoxy-4-nitro-5-sulfophenyl)-2H-tetrazolium-5-carboxanilide
LPS: Lipopolysaccharide
NO: Nitric oxide
iNOS: Inducible nitric oxide synthase
TNF-*α*: Tumor necrosis factor-*α*
IL-1*β*: Interleukin-1*β*
MAPK: Mitogen-activated protein kinase
JNK: c-Jun N-terminal kinase
ERK: Extracellular signal-regulated kinase.
